# Carboxyl-Terminal SSLKG Motif of the Human Cystinosin-LKG Plays an Important Role in Plasma Membrane Sorting

**DOI:** 10.1371/journal.pone.0154805

**Published:** 2016-05-05

**Authors:** Francesco Bellomo, Anna Taranta, Stefania Petrini, Rossella Venditti, Maria Teresa Rocchetti, Laura Rita Rega, Serena Corallini, Loreto Gesualdo, Maria Antonietta De Matteis, Francesco Emma

**Affiliations:** 1 Department of Nephrology-Urology, Division of Nephrology and Dialysis, Bambino Gesù Children’s Hospital and Research Institute, Rome, Italy; 2 Confocal Microscopy Core Facility, Bambino Gesù Children’s Hospital and Research Institute, Rome, Italy; 3 Telethon Institute of Genetics and Medicine (TIGEM), Pozzuoli (NA), Italy; 4 Department of Emergency and Organ Transplantation (DETO), Nephrology, Dialysis and Transplantation Unit, University of Bari Aldo Moro, Bari, Italy; The Chinese University of Hong Kong, HONG KONG

## Abstract

Cystinosin mediates an ATP-dependent cystine efflux from lysosomes and causes, if mutated, nephropathic cystinosis, a rare inherited lysosomal storage disease. Alternative splicing of the last exon of the cystinosin sequence produces the cystinosin-LKG isoform that is characterized by a different C-terminal region causing changes in the subcellular distribution of the protein. We have constructed RFP-tagged proteins and demonstrated by site-directed mutagenesis that the carboxyl-terminal SSLKG sequence of cystinosin-LKG is an important sorting motif that is required for efficient targeting the protein to the plasma membrane, where it can mediate H^+^ coupled cystine transport. Deletion of the SSLKG sequence reduced cystinosin-LKG expression in the plasma membrane and cystine transport by approximately 30%, and induced significant accumulation of the protein in the Golgi apparatus and in lysosomes. Cystinosin-LKG, unlike the canonical isoform, also moves to the lysosomes by the indirect pathway, after endocytic retrieval from the plasma membrane, mainly by a clathrin-mediated endocytosis. Nevertheless, silencing of AP-2 triggers the clathrin-independent endocytosis, showing the complex adaptability of cystinosin-LKG trafficking.

## Introduction

The carboxyl-terminal portion of proteins often contains key sequences that are essential for accurate protein sorting and signaling [1,2]. Moreover, proteins can undergo reversible or irreversible post-translational modifications, producing additional changes at their terminal sequences that modify their biological properties. Changes in the C-terminal sequence can modify temporal and/or spatial distribution of peptides in cells, which often translates into different biological properties of a given protein, according to the cell compartment in which it is expressed, and to the biological state of the cell [3].

In humans, the *CTNS* gene encodes for cystinosin (UniProt # O60931-1), a cystine/H^+^ symporter that mediates the efflux of cystine in the presence of a proton gradient. The protein is predominantly expressed in the lysosomal membrane and is predicted to have seven transmembrane domains [4]. In lysosomes, ATP hydrolysis provides the energy to the V-type ATPase to generate a proton gradient allowing cystine/proton co-transport (molar ratio 1:1) through cystinosin [5]. Mutations in the *CTNS* gene results in massive accumulation of cystine in lysosomes and causes cystinosis (MIM 21980), a rare multisystemic disorder that represents the first cause of renal Fanconi syndrome in early childhood. Essential to the role of cystinosin in cells, sorting of the protein to lysosomes requires at least two targeting motifs, namely a classical tyrosine-based motif (GYDQL), located at the C-terminal end, and a conformational motif (YFPQA), located in the putative fifth inter-transmembrane loop [6].

**Fig 1:**
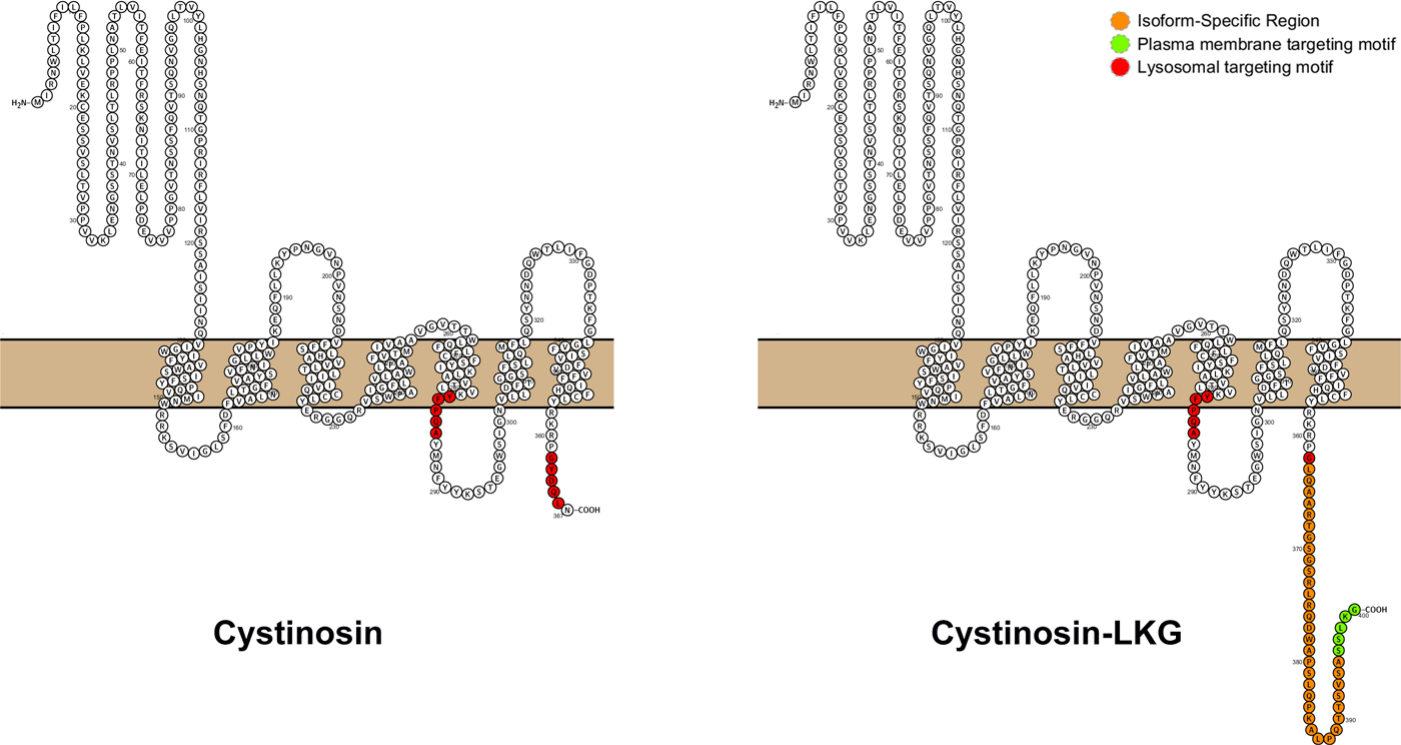
Scheme of the cystinosin isoforms structure. Cystinosin (367 aa) on the left and the cystinosin-LKG isoform (400 aa) on the right, are the main known isoforms to date, for which has been described the transport of cystine. The open-source tool for visualization of proteoforms, PROTTER [32], displayed the hypothetical structure of the two isoforms. Red regions are two targeting motifs for the protein sorting to lysosomes: GYDQL located at the C-terminal end, and YFPQA located in the putative fifth inter-transmembrane loop. Cystinosin-LKG differs from the canonical cystinosin in the C-terminal region (orange) while the proposed motif critical for the protein sorting to the plasma membrane (SSLKG) is highlighted in green.

In addition to the originally described lysosomal protein, we have identified a second isoform (UniProt # O60931-2), termed cystinosin-LKG, based on the sequence of the last amino acids, that is produced by an alternative splicing of exon 12, and which differs from its canonical counterpart only in the carboxyl-terminal sequence (Fig 1). This results in a more scattered expression of the protein in different cell compartments, albeit cystine transport properties do not appear to be modified [7]. Cystinosin-LKG is expressed at high levels in renal tubular epithelia, in the liver, in pancreatic islets of Langerhans, in mucoserous glands of the bronchial epithelia, in melanocytes and in keratinocytes [8]. In the testis, the amount of cystinosin-LKG transcripts matches those of the canonical isoform; cystinosin-LKG is expressed at particularly high levels in Leydig cells [8]. In general, many cells expressing high amounts of cystinosin-LKG have secretory activities, suggesting that this isoform may be important for intracellular trafficking and secretory functions. To date however, these have not been further clarified. A major obstacle is represented by the absence of anti-cystinosin antiserum that specifically recognizes the most abundant isoform that is exclusively expressed in lysosomes. We have been successful in generating a cystinosin-LKG specific antibody, which however is not sensitive enough to analyze the subcellular distribution of the protein.

To overcome these limitations, we have studied the subcellular distribution of RFP-tagged cystinosin-LKG in stably transfected human kidney epithelial cells (HK-2). Our data show that cystinosin-LKG can reach the lysosomal compartment through the constitutive secretory pathway, which directs the protein to the plasma membrane, where it is then retrieved by clathrin-dependent endocytosis and transferred to late endosomes and to lysosomes. Deletion of the SSLKG motif located at the terminal end alters the cell distribution of the protein, indicating that the carboxyl-terminal region plays a pivotal role in cystinosin-LKG sorting.

## Material and Methods

### Cell Cultures

Human Kidney cell line HK-2 (ATCC # CRL-2190) were growth in DMEM F-12 GlutaMax (Gibco) supplemented with 5% fetal bovine serum (Gibco), 100 units/ml penicillin and 100 μg/ml streptomycin, 10 μg/ml insulin from bovine pancreas, 5.5 μg/ml human transferrin (substantially iron-free), and 5 ng/ml sodium selenite (Sigma–Aldrich), in a humidified atmosphere with 5% CO_2_ and 37°C.

### Plasmid Constructs and Transfection

The full-length CTNS-LKG coding sequence was fused in frame at its carboxyl-terminal region with RFP cDNA and inserted into the expression vectors pcDNA 3.1 (Invitrogen). Alternatively, the full-length cDNA encoding cystinosin-LKG was subcloned into pcDNA6/V5His (Invitrogen) or pEGFP-N1 (Clontech) vectors. S396A, S397A, L398A, K399A, G400A, ΔYFPQA and ΔSSLKG deleted mutants were generated by site-directed mutagenesis with Q5 Site-Directed Mutagenesis Kit (New England Biolabs). Sequence integrity was tested by direct sequencing. After plasmid purification, cells were transfected in HK-2 cells using Lipofectamine LTX (Invitrogen), following manufacturer’s instructions. Cells stably overexpressing RFP fusion protein were selected with 3 μg/ml hygromycin B.

### Quantification of RFP-positive cells by flow cytometry

HK-2 transfected with RFP tagged cystinosin-LKG or ΔSSLKG deleted mutant were washed with PBS and harvested in ice-cold PBS-1% FBS to perform flow cytometry on FACSCanto™II flow cytometer with the CellQuest software (BD Biosciences). Dead cells and debris were excluded by gating on forward/side light scatter, and 10,000 events, corresponding to RFP positive cells were analyzed per sample. The intensity level of fluorescence was determined by calculating the geometric mean of fluorescence intensity.

### Immunofluorescence

Stably transfected HK-2 cells expressing RFP-tagged cystinosin-LKG were grown in the 8 wells Chamber Slides (BD Falcon). Cells were fixed in 4% paraformaldehyde for 10 min at 4°C, permeabilized with 0.05% Triton X-100 for 5 min at 4°C, blocked with 1% FBS in PBS for 1 h at room temperature, and stained with primary antibody diluted 1:100 for 1 h at 4°C, followed by two 10 min washes with PBS and incubation with FITC-conjugated anti-mouse IgG antibody for 30 min at 4°C. Lysosomes were stained with anti-LAMP-2 or anti-LAMP-1 mAb (Santa Cruz Biotechnology), endoplasmic reticulum (ER) with anti-PDI mAb (Abcam), Golgi with anti-GM130 mAb (BD Biosciences).

HK-2 transfected with cystinosin-LKG and ΔSSLKG conjugated with RFP or V5His were fixed in 4% paraformaldehyde for 10 min at 4°C, permeabilized 30 min at room temperature with 0.05% saponin, 0.05% BSA, 50 mM NH_4_Cl, blocked with 1% BSA in PBS for 1 h, and stained with anti-RFP (Abcam) or anti-His Tag, clone HIS.H8 (Millipore) diluted 1:200 for 1 h, incubated with FITC-conjugated anti-rabbit or anti-mouse IgG antibody respectively, in alternated washing steps with PBS.

### Confocal Laser Microscopy

The confocal microscopy imaging was performed on Olympus Fluoview FV1000 confocal microscope equipped with FV10-ASW (version 2.0 software), using the 63X oil objective (numerical aperture 1.4). Optical single sections were acquired with a scanning mode format of 1024x1024 pixels, with a pixel size of 0.21 μm sampling, speed of 40 μs/pixel and 12 bits/pixel images. Acquisition of automated-sequential collection of multi-channel images was performed in order to reduce spectral crosstalk between channels. Lasers' power, beam splitters, filter settings, pinhole diameters (equivalent to 1 Airy unit) and scan mode were the same for all examined samples of each staining. Colocalization analysis was performed using ImageJ software downloaded from http://rsb.info.nih.gov/ij (JACoP plugin).

Tables of images were processed using Adobe Photoshop CS6 software (Adobe Systems Inc).

### Live cell imaging and photobleaching experiments

Confocal time-lapse microscopy was performed on a Leica TCS-SP8X laser-scanning confocal microscope equipped with a resonant scanner (Leica Microsystems, Mannheim, Germany), a 488 nm Ar laser, and a stage incubator (OkoLab, Naples, Italy) allowing to maintain stable conditions of temperature, CO_2_ and humidity during live cell imaging. Images were acquired using a HC PLAPO 63X glycerol immersion objective (numerical aperture: 1.30; Leica Microsystems).

For FRAP experiments, 24 hours post-transfection cells were imaged using an LSM710 confocal microscope (Zeiss) fitted with a 488 nm argon and 568 nm ion laser lines, using a 63x PlanApochromat NA 1.4 DIC oil immersion objective. FRAP experiments were performed as following. Briefly, single or multiple region of interest (ROIs) were depicted into the co-expressing cells. Cells were acquired 5 frames before bleaching (4 sec/frame). Bleaching was performed with 100% power of the 488 and 568 lasers for 30 iterations. Recovery was monitored up to 400 seconds after bleaching, using 3% power of the laser. FRAP recovery curves were generated from background subtracted images and the values of fluorescence intensity at each time point were normalized singly to the initial fluorescence as described [9]. Bleaching was minimal during the time course of recovery, between 0–10% and where bleaching exceeded 10%, the recovery sequences were excluded.

For fluorescence loss in photobleaching (FLIP) experiments, a living cell was repeatedly bleached in the same region of interest (ROI) using high laser power and imaging was performed before each new round of bleaching. In particular, cells were imaged before photobleaching with 2% Ar laser power, followed by repeated photobleaching using a single bleach pulse (100% laser power) at intervals of 1 sec for 200 iterations. The loss of fluorescence in a ROI outside of the bleached region was measured over time by LAS AF software.

### Total plasma membrane protein purification

HK-2 cells stably transfected with cystinosin-LKG-RFP were treated with Sulfo-NHS-SS-Biotin for 30’ at 4°C using EZ-Link Sulfo NHS-SS Biotinylation Kit (Pierce). Cells were then processed according to the Manufacturer’s instructions. Briefly, surface-biotinylated cells were lysed; nuclei and whole cells were discarded. Lysates were then purified on streptavidin affinity columns.

### Cystine uptake assay

HK-2 cells stably transfected with cystinosin isoforms or mutant protein were growth in 24-well plates. After washing with Krebs-Henseleit-HEPES buffer (4 mM KCl, 135 mM NaCl, 1 mM MgSO_4_, 1 mM CaCl_2_, 1 mM monobasic NaH_2_PO_4_, 5 mM D-glucose, and 10 mM HEPES, pH 7.4), cells were incubated 20 min at 20°C with 0.1 μCi L-[^14^C]-cystine in 200 μl of Krebs-Henseleit-HEPES buffer, pH 7.4 or pH 5.6. After washing in ice-cold buffer, cells were lysed with 0.1 N NaOH and counted in a Beckman Coulter liquid scintillation counter (Fullerton, CA).

### SDS Gel electrophoresis

Samples were boiled and resolved in 4–12% pre-casted SDS-polyacrylamide gel (Biorad), transferred to nitrocellulose membrane (GE Healthcare), blocked in 5% non-fat dry milk/TBS+0.01% Tween (blocking solution) for 1h. Blotting was performed with rabbit polyclonal anti-human RFP (Abcam) or with mouse monoclonal anti-human Integrin subunit β1 (BD Biosciences) or rabbit polyclonal anti-human AP2 mu1 (GeneTex) or mouse monoclonal anti-human β actin (Ambion). Immunoblots were developed with long lasting chemiluminescence substrate (Euroclone) and acquired with the ChemiDoc™ XRS System (Bio-Rad).

### Transferrin uptake

Transferrin uptake control assays were performed using Alexa-488 labeled human transferrin (Molecular Probes). HK-2 cells were serum starved for 30 min, kept on ice for 10 min, then incubated with 25 μg/ml Alexa-488 transferrin for 15 min at 4°C for binding, washed, and transferred to 37°C for 15 min. When occurred, the endocytosis inhibitors were added during two last steps.

### siRNA Construction and Transfection

Custom siRNA construction was supplied from Thermo Scientific Dharmacon. Labelling of siRNA was performed by using siRNA labelling kit (Ambion Inc.) according to the Manufacturer’s kit. HK-2 cells cultured in 24-well plate were transfected with 200 nM of silencer siRNA oligonucleotides pool directed against human subunit μ1 of AP-2 (*AP2M1*) or with a scrambled negative control oligonucleotide using Oligofectamine™ Reagent (Invitrogen) as suggested by the supplier. Total RNA was extracted from HK-2 cells by using TRIzol® Reagent (Invitrogen); RT-PCR was performed using First Strand cDNA Synthesis Kit (Roche). The primer pair of *AP2M1* (sense: 5’-ATAAGATGTGTGACGTGATGGC-3’; antisense: 3’-CTACTTCTCGATGGACTCACCG-5’) was specific for a 371-bp fragment of *AP2M1* transcripts. The primer pair of glyceraldehyde-3-phosphate dehydrogenase (GAPDH) (sense: 5’-CTGCACCACCAACTGCTTAG-3’; antisense: 3’-AGGTCCACCACTGACACGTT-5’) was specific for a 282-bp fragment of *GAPDH* transcripts. The PCR conditions for *AP2M1* consisted of an initial denaturation at 94°C for 3 min followed by 35 cycles of 45 s at 94°C, 45 s at 56°C, and 45 s at 72°C with a final extension at 72°C for 10 min. The PCR conditions for *GAPDH* consisted of an initial denaturation at 94°C for 3 min followed by 25 cycles of 45 s at 94°C, 45 s at 55°C, and 45 s at 72°C with a final extension at 72°C for 10 min. A 10μl aliquot of the PCR products was electrophoresed on a 1.5% agarose gel following Gelred™ Nucleic Acid Gel Stain (Biotium, Inc.) staining and acquired with ChemiDoc™ XRS System (Bio-Rad).

### Statistical Analysis

Data of colocalization (Pearson’s correlation coefficient, Rr) were obtained by selecting ROIs (Region of Interest) from at least 30 cells and analyzed with JACoP plugin [10] of ImageJ software in three independent experiments and expressed as mean ± SE. P values were estimated by non-parametric, unpaired, two-tailed t-test.

## Results

### Subcellular distribution of the cystinosis-LKG and of cystinosin-LKG mutant

**Fig 2:**
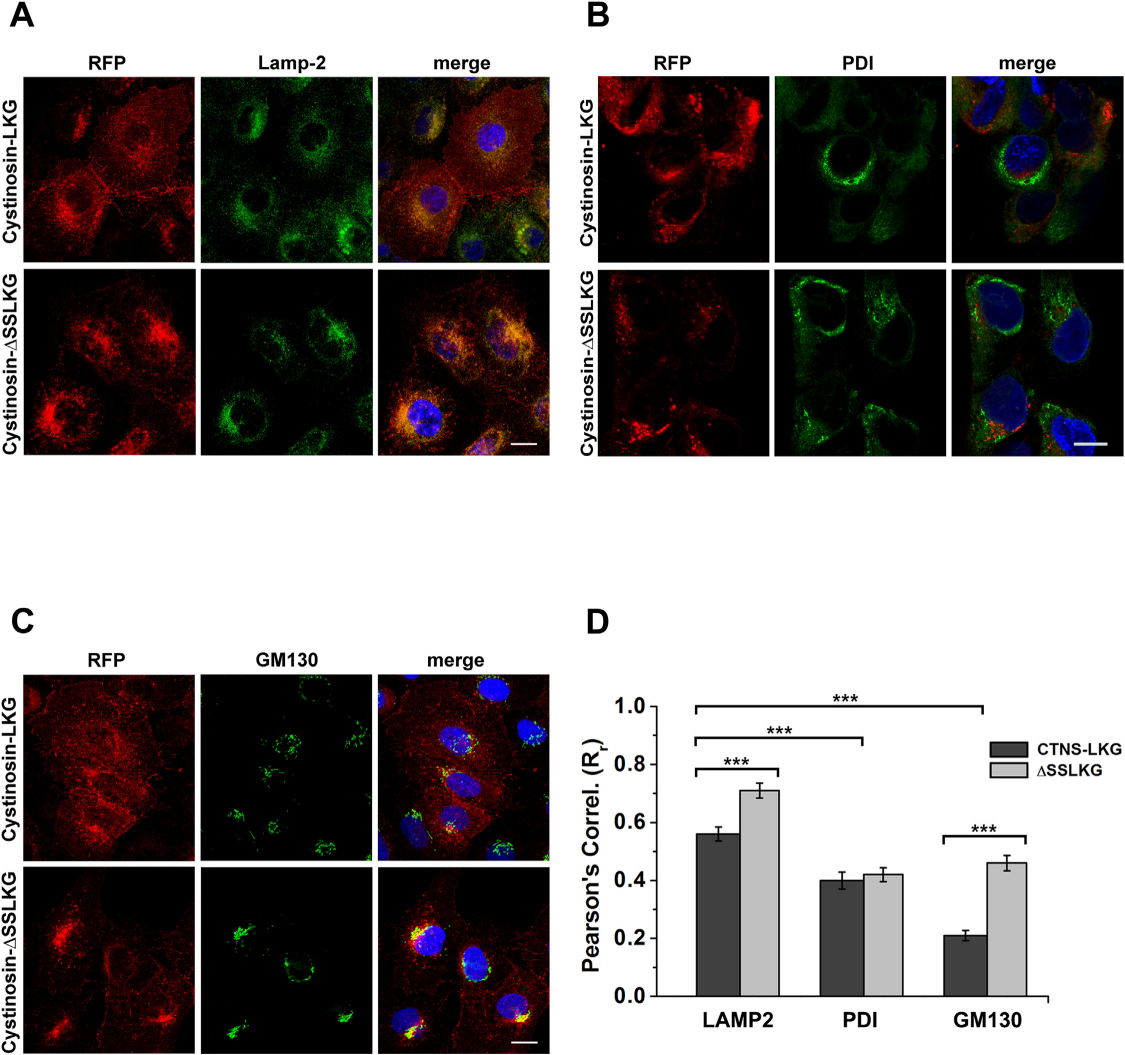
Subcellular distribution of the cystinosin-LKG and ΔSSLKG mutant. HK-2 cells were stably transfected with RFP-tagged cystinosin-LKG or with its mutated form, deleted in C-terminal tail for the last five amino acids (ΔSSLKG). Cells were immunolabeled with LAMP-2 for lysosomes (A), PDI for endoplasmic reticulum (B), GM130 for Golgi (C). Scale bar = 10 μm. Analysis of ROIs (Regions of Interest) shows a Pearson’s Correlation (R_r_) for RFP with LAMP-2 greater than with other organelle markers (*p* < 0.0005). In particular, the mutant ΔSSLKG shows an R_r_ for LAMP-2 significantly increased (*p* < 0.0005) compared to the wild type cystinosin-LKG. The presence of cystinosin-LKG and ΔSSLKG mutant on ER is low, moreover the analysis shows very low expression of cystinosin-LKG in the Golgi apparatus, whereas high R_r_ for GM130 in ΔSSLKG mutant suggests that it is accumulated significantly (*p* < 0.00001) on the Golgi apparatus (D). Means ± SEM of three experiments are shown.

As mentioned above, no isoform-specific antibodies providing accurate subcellular distribution of cystinosin are currently available. To overcome this limitation, we have generated HK-2 cell lines stably transfected with RFP-tagged cystinosin-LKG or RFP-tagged cystinosin-ΔSSLKG that lack the last five amino acid. Immunofluorescence studies (Fig 2A–2C) showed differences in the co-localization and a distribution of RFP-tagged cystinosin-LKG and cystinosin-ΔSSLKG. Pearson’s correlation coefficients (Rr) (Fig 2D), revealed that cystinosin-LKG and cystinosin-ΔSSLKG co-localize more with lysosomal-associated membrane protein 2 (LAMP-2) than with protein disulfide isomerase (PDI) and with the Golgi matrix protein (GM130) (p < 0.0005), indicating higher localization in lysosomes. A moderate but significant increase in lysosomal localization of the ΔSSLKG mutant proteins was observed (Rr_LKG_ = 0.56 ± 0.03 *vs* Rr_ΔSSLKG_ = 0.71 ± 0.03; *p* < 0.0005). Co-localization with ER markers was less prominent and no significant changes were observed between wild-type and ΔSSLKG mutants (Rr_LKG_ = 0.40 ± 0.03 *vs* Rr_ΔSSLKG_ = 0.42 ± 0.02; *p* = NS). In contrast, expression in the Golgi compartment was low for the wild-type protein, but increased very significantly in the ΔSSLKG mutants (Rr_LKG_ = 0.21 ± 0.02 *vs* Rr_ΔSSLKG_ = 0.46 ± 0.03; *p* < 0.00001).

## Determination of expression in the plasma membrane of cystinosin-LKG and cystinosin-ΔSSLKG

**Fig 3:**
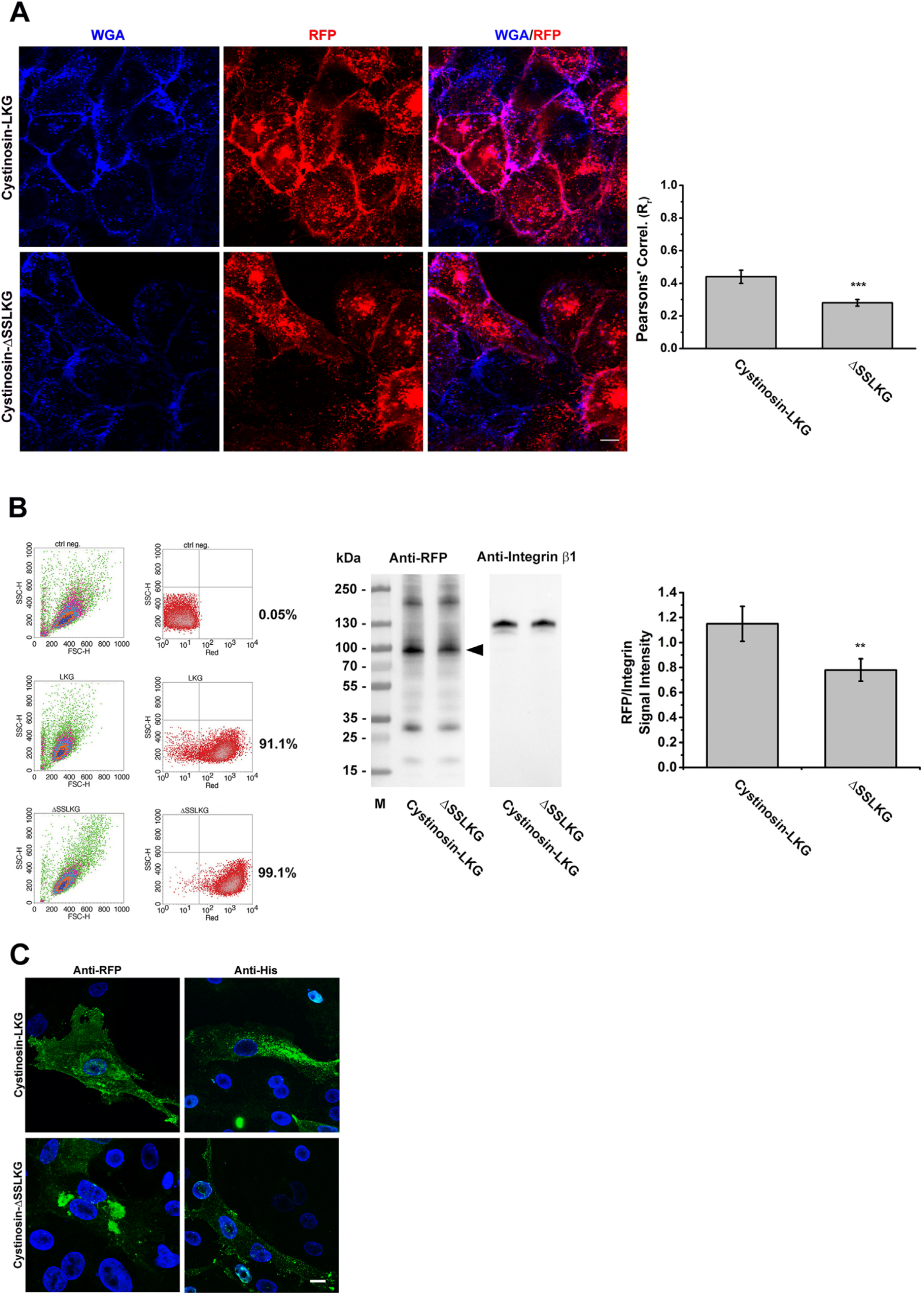
Differential expression of the cystinosin-LKG and ΔSSLKG mutant on the plasma membrane. HK-2 cells stably transfected with RFP-tagged cystinosin-LKG or ΔSSLKG were stained with WGA Blue to mark the plasma membrane. Colocalization analysis shows that deletion of -SSLKG motif reduces about 35% (*p* < 0.001) expression of the carrier on the plasma membrane (A). Scale bar = 10 μm, means ± SEM of four experiments are shown. Stably transfected cells with cystinosin-LKG or ΔSSLKG mutant were analyzed by cytofluorimetry to define the transfection efficiency, and a cell surface protein biotinylation for SDS-PAGE analysis was performed. Quantitative analysis by evaluation of RFP signal normalized with Integrin β1 shows about 30% reduction (*p* < 0.01) in ΔSSLKG mutant (B). Means ± SEM of three experiments are shown. HK-2 cells transiently transfected with RFP- or V5His-tagged cystinosin-LKG and ΔSSLKG were immunolabeled using anti-RFP/ Alexa Fluor® 488 goat anti-rabbit IgG antibody or anti-His Tag/Alexa Fluor® 488 goat anti-mouse IgG antibody, respectively (C). Scale bar = 10 μm.

Changes in the expression of cystinosin-LKG in the plasma membrane were assessed by co-localization experiments using blue wheat germ agglutinin as a membrane marker (Fig 3A). Semi-quantitative analysis by confocal microscopy revealed a significant reduction of the ΔSSLKG mutant protein at the cell surfaces (Rr_LKG_ = 0.44 ± 0.04 *vs* Rr_ΔSSLKG_ = 0.28 ± 0.02; *p* < 0.001). This result was confirmed by cell surface biotinylation followed by SDS-PAGE analysis of biotinylated proteins. The plasma membrane expression of cystinosin-LKG was reduced by approximately 30% after deleting the SSLKG sequence. This effect was not related to differences in the levels of expression of proteins, as assessed by flow cytofluorimetry (91.1% RFP positive for cystinosin-LKG *vs* 99.1% of ΔSSLKG mutant) (Fig 3B). To verify that the fusion of the RFP to the C terminus of cystinosin-LKG and the ΔSSLKG mutant did not interfere with their localization, an additional construct was made by using a V_5_His tag in frame with the protein sequences. Immunofluorescence studies using the anti-RFP antibody confirmed that the fluorescent signal did indeed correspond to RFP tagged cystinosin-RFP. Immunofluorescence with anti-His Tag on HK-2 transiently transfected with V5His-tagged cystinosin-LKG and ΔSSLKG mutant showed a distribution similar to the RFP-tagged proteins (Fig 3C).

Site-directed mutagenesis was performed to identify critical amino acid residues in the SSLKG motif. Overall, all substitutions limited to one single amino acid of the SSLKG motif did not alter significantly the membrane distribution of the protein; substitution of serine at position 397 with alanine showed some variability in the distribution of cystinosin-LKG, with a moderate decrease in the expression observed in some cells, but this finding was not uniform (S1 Fig).

Culture conditions that stimulate expression of cystinosin-LKG, like serum starvation and medium without methionine and cystine, dramatically increased the expression of the cystinosin-LKG and of the ΔSSLKG mutant in all compartments (S2 Fig). In contrast, the expression of the more abundant lysosomal isoform was limited to lysosomal structures.

### ^14^C-Cystine uptake in HK-2 stably transfected with the cystinosin isoforms or ΔSSLKG mutant

**Fig 4:**
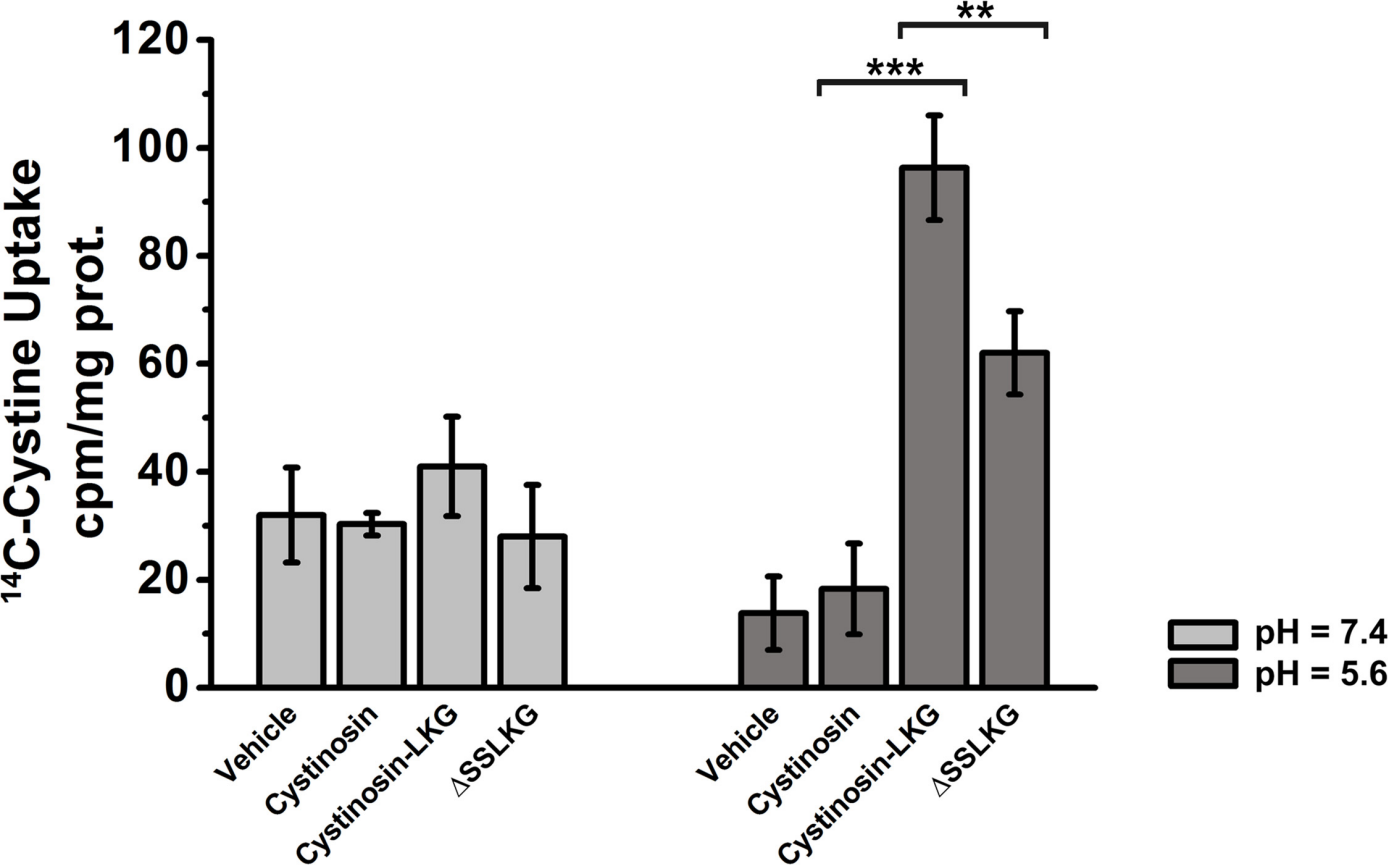
^14^C-cystine uptake from extracellular milieu in HK-2 transfected with different cystinosin isoforms. ^14^C-cystine uptake assay performed in HK-2 cells transfected with vehicle, cystinosin, cystinosin-LKG, or ΔSSLKG mutant, shows low uptake of radiolabelled L-[^14^C]-cystine across plasma membrane in absence of a proton gradient (light grey columns). In the presence of a proton gradient (dark grey columns) HK-2 cells overexpressing cystinosin-LKG show a significant ^14^C-cystine uptake (p < 0.0005) compared to cells transfected with cystinosin; while HK-2 cells overexpressing ΔSSLKG mutant show lower ^14^C-cystine uptake (p < 0.004) compared to cells transfected with cystinosin-LKG. Means ± SEM of three experiments are shown.

In order to verify if differences in the plasma membrane expression of cystinosin-LKG and cystinosin-ΔSSLKG had an impact on cystine uptake from the extracellular milieu radiolabelled L-[^14^C]-cystine assays were performed. As shown in Fig 4, in the absence of a proton gradient (pH = 7.4), overexpression of cystinosin, cystinosin-LKG and cystinosin-ΔSSLKG had no impact on L-[^14^C]-cystine uptake. However, in the presence of a proton gradient (pH = 5.6), uptake of radiolabelled L-[^14^C]-cystine was significantly increased in HK-2 stably transfected with cystinosin-LKG compared to the untransfected cells (*p* < 0.00005) and stably transfected with RFP-tagged cystinosin (*p* < 0.0005). The uptake of L-[^14^C]-cystine in HK-2 cells stably transfected with cystinosin-ΔSSLKG was significantly reduced by approximately 35% (*p* < 0.004), a results that is compatible with the different degree of expression of these two proteins in the plasma membrane.

### Cystinosin-LKG dynamics in living HK-2

**Fig 5:**
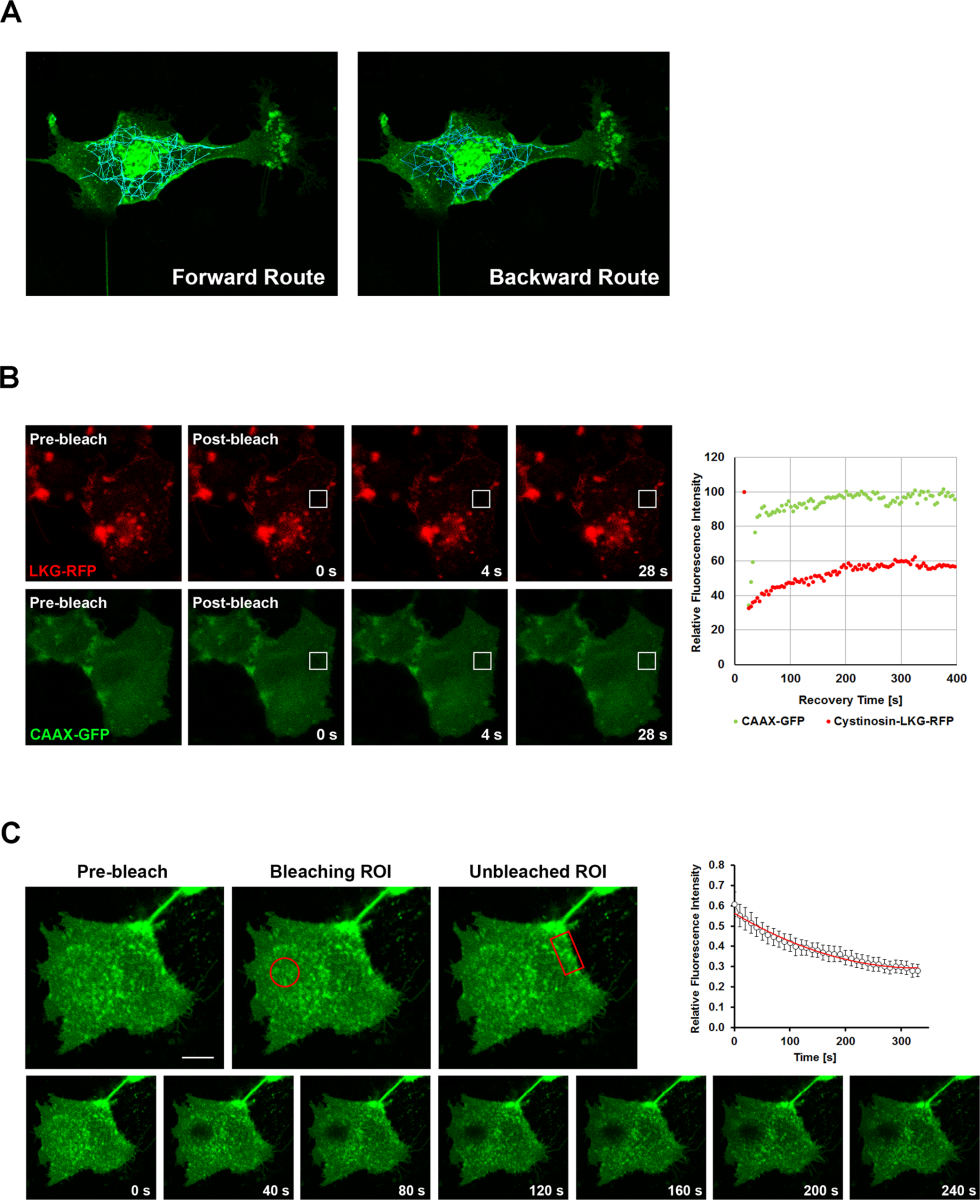
Tracking, FRAP and FLIP analysis in HK-2 transfected with cystinosin-LKG. HK-2 cells were transiently transfected with EGFP tagged cystinosin-LKG. Tracking of fluorescent vesicles with an estimated diameter > 1 micron, showed the centrifugal (left) and centripetal (right) movements (A). Dynamics of cystinosin-LKG on the plasma membrane was investigated by FRAP (Fluorescence Recovery After Photobleaching). HK-2 cells were transiently transfected with RFP tagged cystinosin-LKG and GFP-CAAX domain as unrelated control protein. Pre-bleach, bleach and post-bleach images are shown of a representative FRAP, and the recovery over time of the bleached region of interest (ROI) is graphically represented. Analysis of these data suggests that about 36% of cystinosin-LKG on the plasma membrane is represented by a mobile fraction (the percentage of maximally recovered fluorescence) with a mean half-time of recovery measuring t_half_ = 92.2 ± 12.6 s, the time in which 50% of the fluorescence in the bleach spot was recovered (B). To verify if cystinosin-LKG moves from the cytoplasm or lateral boundary, a bleach region of interest (oval) was placed in the cytoplasmic region near plasma membrane and a decay of signal (Fluorescence Loss In Photobleaching–FLIP) was observed only on specific portions of the plasma membrane (rectangle) (C), other regions are not influenced by bleaching. Scale bar = 10 μm, Means ± SEM of four experiments are shown.

Since cystinosin-LKG, differently from the canonical cystinosin, shows a variable subcellular distribution and a peculiar presence in the plasma membrane, we performed confocal live cell imaging, which helps to analyze dynamics of this isoform (S1 Movie). Analysis with TrackMate v. 2.8.2, a plugin of ImageJ, showed the movements of EGFP tagged cystinosin-LKG, embedded into vesicles, towards the plasma membrane and backwards (Fig 5A). Resident and mobile moieties of cystinosin-LKG were assayed by Fluorescence Recovery After Photobleaching (FRAP) of HK-2 transfected with RFP tagged cystinosin-LKG and CAAX-GFP used as internal control. Data analysis indicated that 36.1% ± 4.9 SE is mobile (M_f_), whereas (63.9% ± 4.9 SE) are resident in the plasma membrane (I_f_). After photobleaching RFP tagged cystinosin-LKG fluorescence in the plasma membrane recovers with a mean half-time of recovery (t_half_) = 92.2 ± 12.6 seconds (Fig 5B). To exclude that fluorescence recovery was only due to the contribution of lateral mobility of the cystinosin-LKG, and to assess whether the protein moves from the cytoplasm to the plasma membrane, a complementary approach with Fluorescence Loss in Photobleaching (FLIP) was performed. Repeated bleaching of the same region close to the plasma membrane revealed signal decay of EGFP tagged cystinosin-LKG on the portion of the plasma membrane overlooking the bleach region, demonstrating the anterograde transport of the carrier (Fig 5C).

### Inhibition of the clathrin- or caveolin-dependent endocytosis

**Fig 6:**
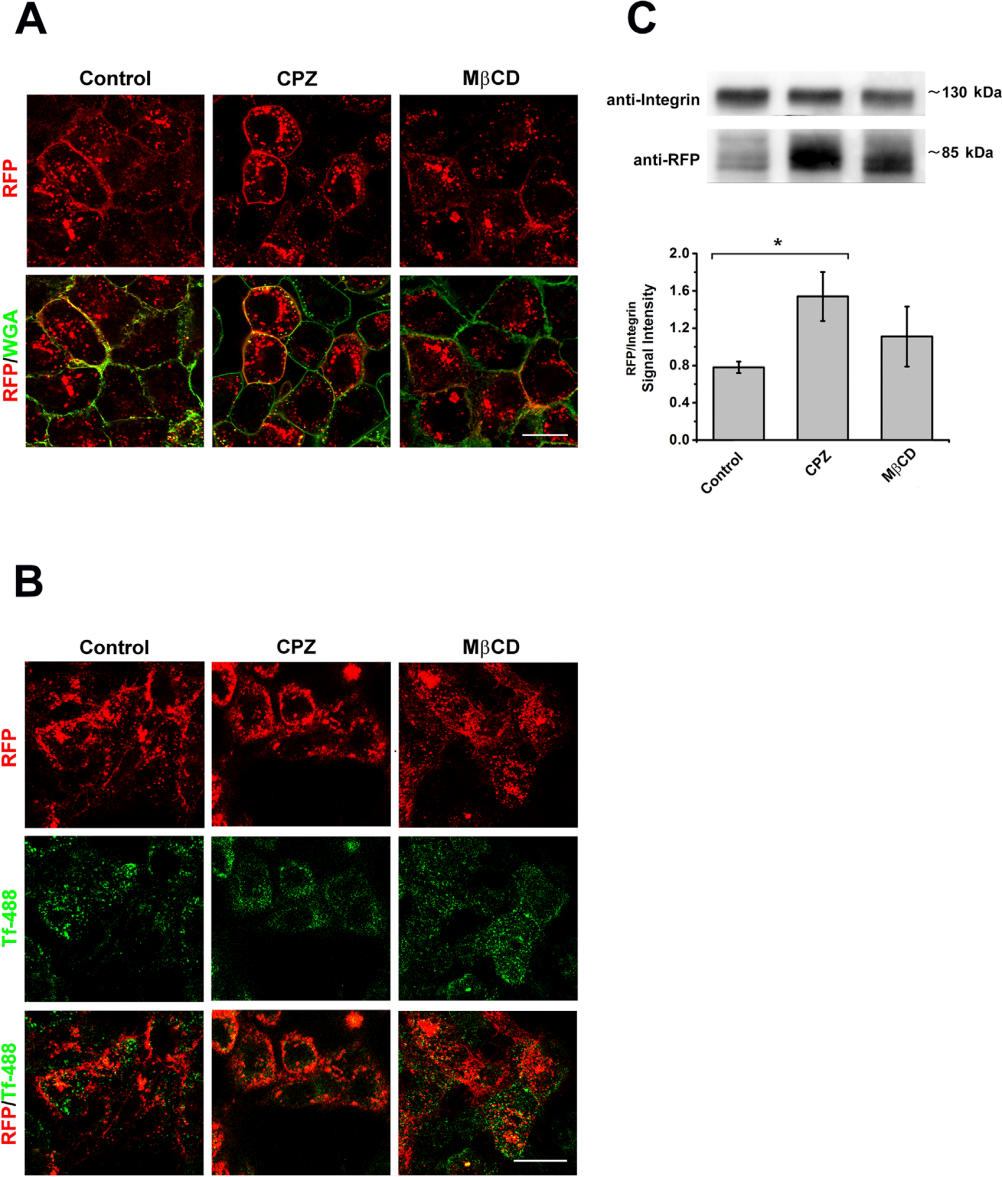
Effect of endocytosis inhibitors on endosomal sorting of cystinosin-LKG. HK-2 cells stably transfected with RFP-tagged cystinosin-LKG, after 48h serum starvation, were treated 30’ with 5 μg/ml chlorpromazine (CPZ) a clathrin-dependent endocytosis inhibitor or with 30 μg/ml methyl-β-cyclodextrin (MβCD) which affects clathrin-independent pathway. Qualitative analysis shows the presence of RFP-tagged cystinosin-LKG on the plasma membrane stained with WGA green, but the RFP signal accumulated more in CPZ treated cells (A). Scale bar = 20 μm. In the same experimental conditions, the uptake of Alexa Fluor® 488 transferrin (488-Tf) was assayed in order to confirm the inhibition of clathrin-dependent endocytosis (B). Scale bar = 20 μm. Quantitative analysis, achieved by protein surface biotinylation and SDS-PAGE, shows that CPZ treatment induces a significant increase of cystinosin-LKG presence on the plasma membrane (C). Means ± SEM of three experiments are shown.

To characterize the mechanisms regulating the endocytic retrieval of cystinosin-LKG from the plasma membrane, we examined in HK-2 cells the effects of selective inhibitors of the clathrin- or caveolin-dependent endocytosis. HK-2 cells, stably transfected with cystinosin-LKG conjugated with RFP, were serum starved for 48 hours. Cells were then treated for 30 min at 37°C with 5 μg/ml chlorpromazine (CPZ), a cationic amphiphilic drug. This drug inhibits clathrin-coated pit formation by reversible removal of clathrin molecules and of the adapter protein complex from the plasma membrane [11]. Alternatively, cells were treated with 30 μg/ml methyl-β-cyclodextrin (MβCD), which depletes membranes from cholesterol and inhibits clathrin-independent pathways. Significant accumulation of cystinosin-LKG in plasma membrane was observed with CPZ treatment, while no significant changes were observed in cells treated with MβCD (Fig 6A). The efficacy of treatments was supported by measurements of transferrin uptake that was specifically blocked by CPZ and not by MβCD (Fig 6B). Cell surface protein biotinylation and subsequent SDS-PAGE analysis confirmed the microscopy data (Fig 6C). Treatment with inhibitors of endocytosis did not affected significantly the presence of cystinosin-LKG in lysosomes (data not shown).

### Silencing of the AP-2 Adaptor Complex

**Fig 7:**
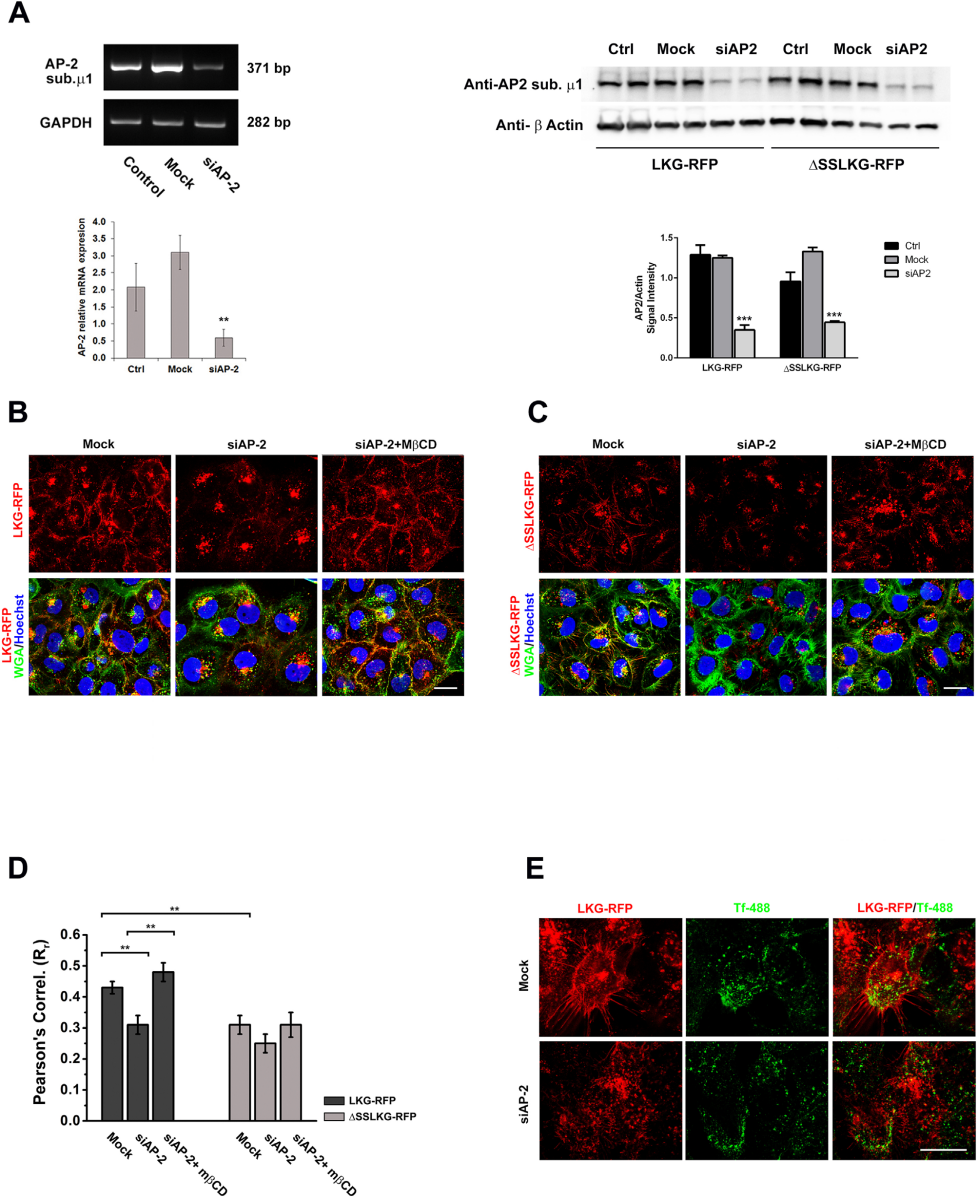
Effect of silencing of AP-2 mu chain on endosomal sorting of cystinosin-LKG and ΔSSLKG mutant. siRNA to human AP-2 mu chain and a scrambled control were transfected into HK-2 cells overexpressing RFP tagged cystinosin-LKG and ΔSSLKG mutant. The analysis of the AP-2 expression by PCR and western blotting show an efficient silencing with a significant transcript reduction (p < 0.005) (A). After 72h silencing of AP-2 mu chain, expression of cystinosin-LKG on the plasma membrane is significantly reduced. Inhibition of the clathrin-independent pathway by treatment with 30 μg/ml methyl-β-cyclodextrin (MβCD) for 30’ combined to AP-2 silencing, permanently prevents endocytic sorting of cystinosin-LKG from the plasma membrane, triggering the accumulation of the protein on the plasma membrane (B). Scale bar = 20 μm. As previously showed, ΔSSLKG mutant is less expressed on the plasma membrane, and this condition is highlighted by AP-2 silencing. After AP-2 silencing, in fact, ΔSSLKG is very low on plasma membrane and the inhibition of the clathrin-independent pathway with MβCD does not affect significantly the distribution (C). Scale bar = 20 μm. Colocalization analysis between RFP and WGA green (plasma membrane) signals shows the Pearson’s Correlation significantly reduced in ΔSSLKG cells (*p* < 0.001) and in cystinosin-LKG with AP-2 silencing (*p* < 0.001). In the latter, after MβCD treatment, the Pearson’s Correlation increases about 24% (*p* < 0.001), while no significant effects is observed in ΔSSLKG mutant (D). Analysis of transferrin uptake indicates that cystinosin-LKG is expressed on the plasma membrane only in cells where AP-2 was not silenced (transferrin internalized) instead silencing of AP-2 associated to the accumulation of transferrin on the plasma membrane, affects negatively the presence of cystinosin-LKG on the plasma membrane (E). Scale bar = 20 μm. Means ± SEM of four (A, B, C, D) or three (E) experiments are shown.

The AP-2 adaptor complex is generally required for the formation and endocytosis of clathrin-coated vesicles. We assessed the role of AP-2 by siRNA interference of the AP-2 mu chain in HK-2 overexpressing RFP-tagged cystinosin-LKG or ΔSSLKG mutant. Transfection efficiency was assessed by PCR showing transcript reduction of about 80% and by western blotting showing protein reduction of about 70% (Fig 7A). Silencing of AP-2 in WGA green stained HK-2 overexpressing cystinosin-LKG significantly reduced the presence of the protein in the plasma membrane compared to the scrambled control (Rr_Mock_ = 0.43 ± 0.02 *vs* Rr_siAP-2_ = 0.31 ± 0.03; *p* < 0.001) (Fig 7B).

Thirty minutes incubation with MβCD, which inhibits clathrin-independent endocytosis, caused a significant accumulation of cystinosin-LKG in the plasma membrane of cells silenced for AP-2 (Rr_siAP-2_ = 0.31 ± 0.03 *vs* Rr_siAP-2+MβCD_ = 0.48 ± 0.03; *p* < 0.001), as shown in Fig 7B. As expected, cystinosin-ΔSSLKG was less abundantly expressed in the plasma membrane of WGA green-stained HK-2 cells. AP-2 silencing and MβCD treatment did not induce statistically significant changes (Rr_Mock_ = 0.31 ± 0.03 *vs* Rr_siAP-2_ = 0.25 ± 0.03; *p* = NS, Rr_siAP-2+MβCD_ = 0.31 ± 0.04; *p* = NS) (Fig 7C). Pearson’s Correlation of different experimental conditions was reported in Fig 7D. The specificity of this result was sustained by the uptake of Alexa-488 transferrin assays, which showed that cells silencing AP2 caused retention of transferrin close to the plasma membrane (Fig 7E).

Silencing of AP-1, which mediates protein sorting from the trans-Golgi network (TGN) to endosomes and backwards, and silencing of AP-3, which plays an important role in the transport of proteins through the endo-lysosomal pathway, had no significant effect on the plasma membrane expression of cystinosin-LKG (Rr_Mock_ = 0.45 ± 0.02 *vs* Rr_siAP-1_ = 0.37 ± 0.04 and Rr_siAP-3_ = 0.46 ± 0.03; *p* = NS) (data not shown).

### Deletion of the Lysosomal-targeting Signal

**Fig 8:**
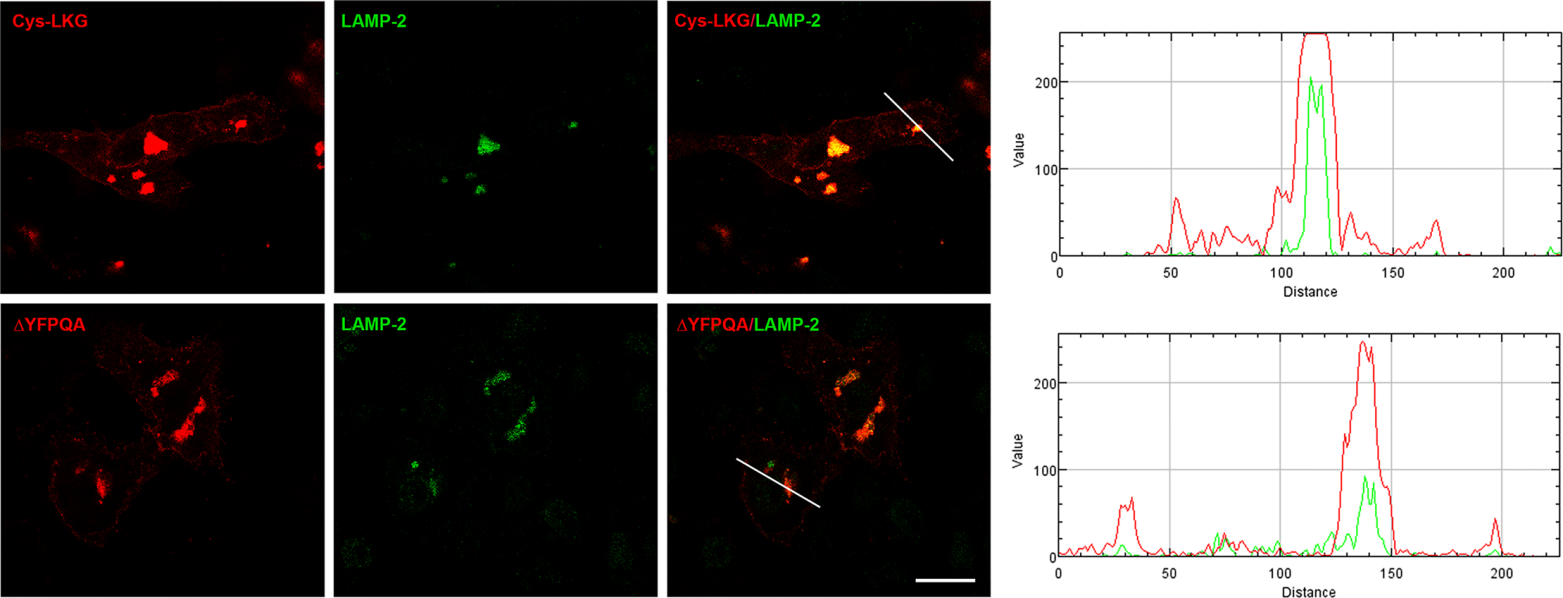
Colocalization studies of cystinosin-LKG carrying the deletion of lysosomal sorting signal YFPQA. In HK-2 cells, transiently transfected with the pCTNS-LKG-RFP construct carrying the deletion of the (ΔFPQA), the deleted cystinosin-LKG-RFP could be seen on the plasma membrane as well as in lysosomes. The intensity profile, obtained with RGB Profiler, an ImageJ plugin, showed the RFP signal in lysosomes of ΔFPQA reduced compared to the wild type cystinosin-LKG. Scale bar = 20 μm.

Cherqui et al. has shown partial relocalization of cystinosin to the plasma membrane after delating the GYDQL lysosomal targeting motif at the carboxy-terminal end and nearly complete relocalization after additional deletion of the YFPQA motif in the 5^th^ inter-transmembrane loop [6]. To assess if partial localization of the LKG isoform to the lysosome was not mediated by the YFPQA motif, we have generated RFP tagged cystinosin-LKG lacking the YFPQA sequence. As shown in Fig 8, this mutated isoform retain the ability to localized to intracellular vesicles that were stained by LAMP-2, indicating that the SSLKG sequence is *per se* able to direct the protein to the lysosomal compartment (Fig 8).

## Discussion

Most recent studies on nephropathic cystinosis have focused on the role and function of the canonical cystinosin isoform that is exclusively expressed in lysosomes. Cystinosin-LKG maintains cystine transport characteristics and follows transcriptional modulation similar to the lysosomal isoform [7,12]. It differs from cystinosin by its less selective subcellular distribution. The functional relevance of this isoform is yet unknown. It is however, largely demonstrated that alternative splicing represents a general mechanism for modulating protein expression and functions [13]. Soluble lysosomal proteins are sorted to the lysosomes by recognition of cleavable N-terminal signal peptides and mannose-6-phosphate residues. Similarly, lysosomal transmembrane proteins share canonical and non-canonical motifs that are required to direct them to the lysosomal membrane. These signals include dileucine-based motifs (DXXLL or [DE]XXXL[LI]) and tyrosine-based motifs (YXXØ), which both interact with components of clathrin coats, such as GGAs (Golgi localized γ-ear containing ARF-binding proteins) and adaptor protein complexes [14].

Cherqui et al. have shown that the GYDQL motif is essential to direct cystinosin to the lysosome, and that combined mutations of the C-terminal sequence and of the 5^th^ putative transmembrane loop redirect nearly entirely the protein to the plasma membrane [6]. Andrzejewska et al. demonstrated that the GYDQL motif has strong AP-3-binding activity, which mediates direct intracellular targeting of cystinosin to late endosomes/lysosomes [15]. Although cystinosin-LKG lacks the tyrosine-based motifs (GYDQL) at its terminal end, it retains the ability to reach the lysosomes. Furthermore, cystinosin-LKG also localizes to other cytosolic organelles and is expressed in the plasma membrane. Most likely, other non-canonical amino acid sequences are responsible for these differences, as also shown for other lysosomal proteins [16]. Herein, we have shown that the amino acidic sequence of the C-terminal region influences the cell expression.

Using reporter molecules and site-directed mutagenesis we have confirmed that the SSLKG sequence is essential to the subcellular distribution of cystinosin-LKG. This motif however, even if could be recognized as a KKXX-like motif, does not act as a signal to transfer the protein from the Golgi apparatus to the ER, like in other proteins harboring dilysine motifs [17]. In fact, we observed very limited expression of cystinosin-LKG in the ER. Conversely, when the SSLKG domain was deleted, the protein accumulated in the Golgi, suggesting that quality control mechanisms of the protein synthesis apparatus prevented expression of the mutant protein somewhere else in the cell [18]. Expression at the plasma membrane level of this isoform was also significantly affected by the deletion of the SSLKG sequence, indicating that sorting and retrieval from the plasma membrane is a physiological step of the normal routing of cystinosin-LKG. The presence of an RFP tag did not influence the relative expression of cystinosin-LKG and ΔSSLKG mutant on the plasma membrane.

It has been well established that lysosomal targeting of lysosomal membrane proteins (LMPs) can follow a direct pathway that transfers newly synthesized proteins from the trans-Golgi network (TGN) directly to the endosomal system [19], or an indirect pathway. In the latter case, proteins are transferred from the TGN to the plasma membrane and subsequently delivered to lysosomes *via* the endocytic pathway [20]. FRAP and FLIP experiments, and more striking live cell imaging of EGFP tagged cystinosin-LKG, show that this isoform moves directionally through the cytoplasm to the plasma membrane and backward. FRAP analysis, moreover, showed that the plasma membrane-associated cystinosin-LKG has a relatively high immobile fraction (63.9 ± 4.9%), that might be due to protein clustering, interactions with scaffolding or cytoskeleton proteins, and/or protein-lipid interactions. Hypothetically, cystinosin-LKG in the plasma membrane could mediate cystine uptake from the extracellular milieu in conditions where cells lack cystine. This would however, require a proton gradient. Heavy expression in the plasma membrane of cystinosin-LKG in cells that are cultured in cystine-free medium supports this item.

This condition, which recalls to the mechanism of proton/substrate coupling fully explained in Ruivo et al. for canonical cystinosin [21], is particularly interesting as physiological aspect; in fact, cystinosin-LKG, in particular conditions, could implements the cystine/glutamate antiporter system x^-^_c_ activity, which is pH sensitive and inhibited by acidic environment [22].

A limited number of lysosomal transport proteins have been well characterized to date [23]. The processing of other integral lysosomal membrane proteins, such as the lysosomal acid phosphatase Battenin (encoded by the gene CLN3, which causes neuronal ceroid lipofuscinoses when mutated) or the mucolipin subfamily of transient receptor potential proteins (TRPMLs, mucolipidosis-associated) is in many aspects similar to cystinosin-LKG, involving passage through the plasma membrane [24–26]. Each protein pathway and the requirements for specific adaptor protein (AP) complexes can differ wildly among lysosomal membrane proteins. In general however, when protein are translocated via the direct pathway AP-1 and/or AP-3 complexes are required, whereas trafficking through the indirect pathway is usually mediated by AP-2 complexes.

Accumulation of cystinosin-LKG in the plasma membrane after inhibition of endocytosis, in particular after inhibition of the clathrin-mediated pathway, without significant changes of the expression in lysosomes, indicates that this isoform reaches lysosomes, at least in part, through the indirect pathway. The observation that cystinosin-LKG can reach lysosomes even in absence of the second unconventional targeting signal located in the fifth inter-TM loop (ΔFPQA mutant) indicates that lysosomal targeting of the LKG isoform is not affected by this motif like the canonical cystinosin.

Surprisingly, silencing of AP-2 did not increases the expression of cystinosin-LKG in the plasma membrane, although the internalization of transferrin (Tf), a widely used marker of clathrin-mediated endocytosis, was highly impaired in these cells. This suggests that, even if AP-2 is essential for the endocytic retrieval mediated by clathrin coated-pit and for the formation of coated-vesicles, the cell can up-regulate alternative endocytic structures to potentiate its trafficking pathways from the plasma membrane [27]. We speculate that a similar mechanism could apply to cystinosin-LKG. Consistent with this hypothesis, combined blockade of the clathrin-dependent pathway with siAP-2 and of the clathrin-independent pathway with MβCD produced accumulation of cystinosin-LKG in the plasma membrane. Whereas, several non-canonical roles for AP2 have begun to emerge. A recent study shows, in fact, that the glutamate receptor GLR-1, similarly to cystinosin-LKG, does not accumulate at the plasma membrane in AP-2 mutants [28].

Overall, our results indicate that cystinosin-LKG is transferred at least in part to the plasma membrane by the secretory pathway, and is subsequently directed to lysosomes after internalization and trafficking through the endosomal system. The SSLKG motif is essential to regulate this process. Post-translational modifications such as cleavage and/or phosphorylation could be critical for modulating protein sorting. Studies are underway and indicate that this process is complex.

At this stage, it is yet unknown if the -LKG isoform has a specific function in secretory vesicles, endosomes and in the plasma membrane, or if detection of the protein in these organelles is simply related to its intracellular trafficking characteristics. More generally, this isoform has been well preserved throughout evolution [7]. It is unknown if it represents a vestigial residue or if it has specific functions in cells that are different from the purely lysosomal isoform. This is particularly relevant since gene therapies could be attempted based on encouraging results obtained in mouse models of bone marrow transplantation in cystinosis [29]. Moreover, increasing evidence shows that cells can transfer material between them; predictably, the lysosomal isoform could be transferred from a wild-type cell to a cystinotic cell through exosomal secretion, while cystinosin-LKG can also be transferred through shedding of microvesicles from the plasma membrane [30,31].

## Supporting Information

S1 Fig Subcellular localization of cystinosin-LKG after site-directed mutagenesis of SSLKG motif. HK-2 cells were transiently co-transfected with CAAX-GFP and cystinosin-LKG-RFP mutated in its C-terminal tail. Substitution of each residue of SSLKG motif with an Alanine did not change significantly the co-localization with CAAX-GFP on the plasma membrane. Scale bar = 10 μm.
(TIF)

S2 Fig Effect of cystine deprivation on the expression of the cystinosin isoforms. HK-2 cells transfected with RFP-tagged cystinosin, cystinosin-LKG or ΔSSLKG mutant were growth for 48 hours in three different conditions: complete medium, serum starvation, CySS/Met free medium and serum starvation. Nutrient deprivation, and in particular redox unbalance due to absence of cystine in the medium, induces a general increase of the isoforms expression in all compartments; these experimental conditions exacerbate the differences in subcellular distribution between cystinosin isoforms. Scale bar = 20 μm.
(TIF)

S1 Movie Confocal live cell imaging of HK-2 transfected with EGFP-tagged cystinosin-LKG. 4x speed movie shows trafficking of cystinosin-LKG-EGFP towards plasma membrane and backwards.
(AVI)
