# Supplementary figures and images for: Carboxyl-Terminal SSLKG Motif of the Human Cystinosin-LKG Plays an Important Role in Plasma Membrane Sorting
Source: PLoS One. 2016 May 5;11(5):e0154805. doi: 10.1371/journal.pone.0154805 (PMC4858208; doi:10.1371/journal.pone.0154805)

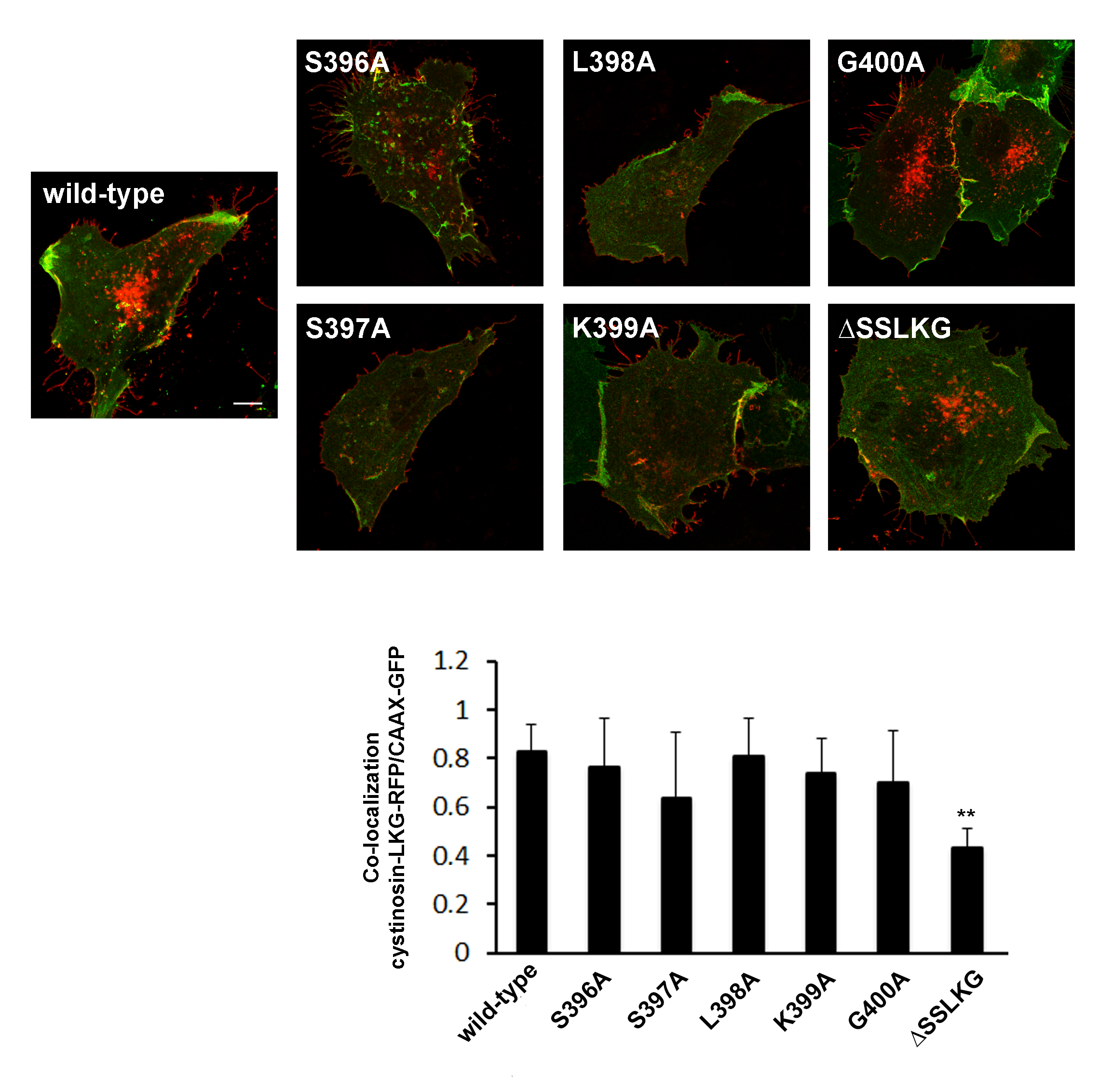

Supplement: S1 Fig — HK-2 cells were transiently co-transfected with CAAX-GFP and cystinosin-LKG-RFP mutated in its C-terminal tail. Substitution of each residue of SSLKG motif with an Alanine did not change significantly the co-localization with CAAX-GFP on the plasma membrane. Scale bar = 10 μm. (TIF) [file pone.0154805.s001.tif]

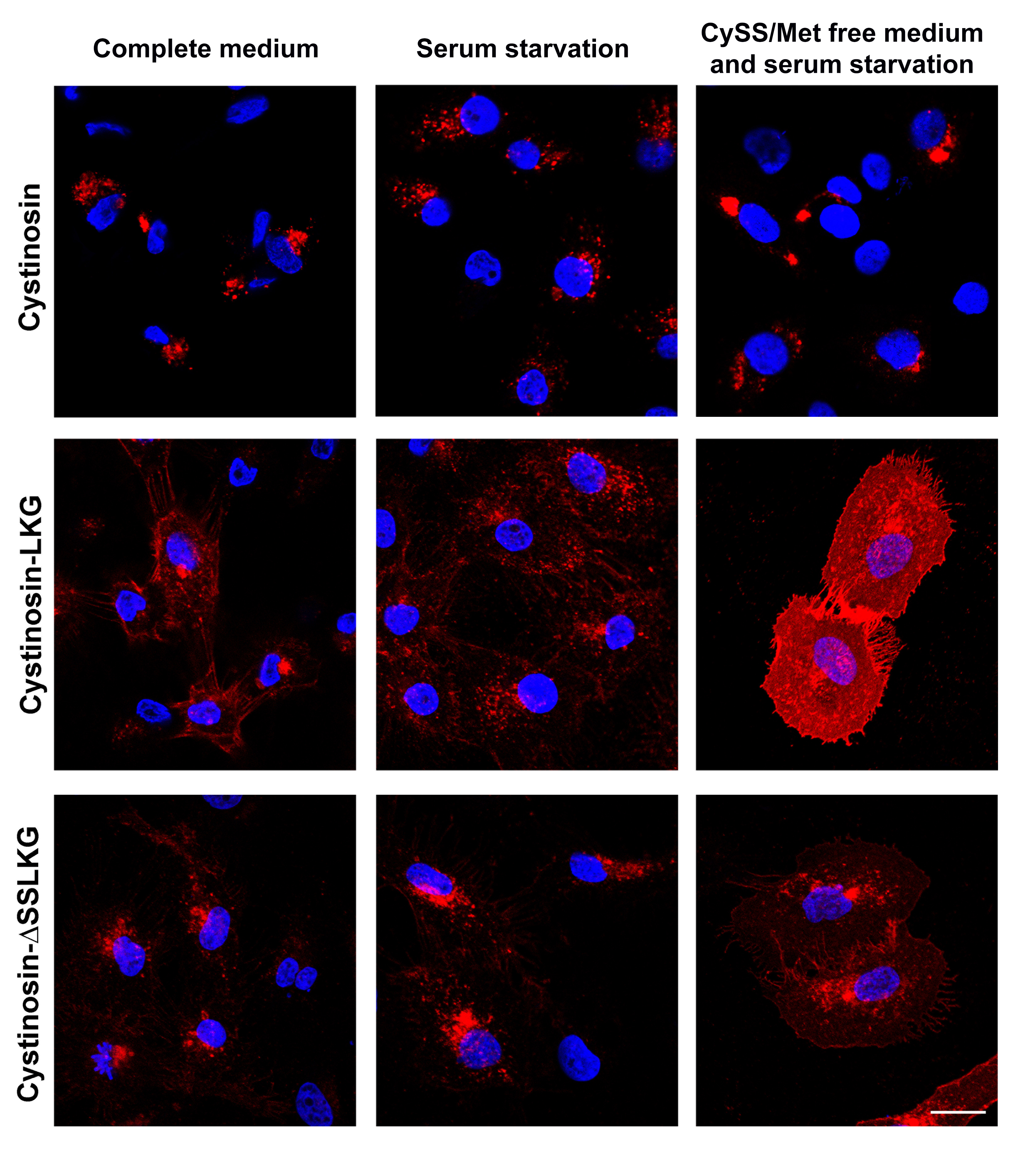

Supplement: S2 Fig — HK-2 cells transfected with RFP-tagged cystinosin, cystinosin-LKG or ΔSSLKG mutant were growth for 48 hours in three different conditions: complete medium, serum starvation, CySS/Met free medium and serum starvation. Nutrient deprivation, and in particular redox unbalance due to absence of cystine in the medium, induces a general increase of the isoforms expression in all compartments; these experimental conditions exacerbate the differences in subcellular distribution between cystinosin isoforms. Scale bar = 20 μm. (TIF) [file pone.0154805.s002.tif]
